# High throughput quantification of apolipoproteins A‐I and B‐100 by isotope dilution MS targeting fast trypsin releasable peptides without reduction and alkylation

**DOI:** 10.1002/prca.201600128

**Published:** 2017-04-03

**Authors:** Bryan A Parks, David M Schieltz, Michael L Andrews, Michael S Gardner, Jon C Rees, Christopher A Toth, Jeffrey I Jones, Lisa G McWilliams, Zsuzsanna Kuklenyik, James L Pirkle, John R Barr

**Affiliations:** ^1^ Division of Laboratory Sciences Centers for Disease Control and Prevention Atlanta GA USA

**Keywords:** Apolipoproteins, Cardiovascular system, Mass Spectrometry, LC‐MS/MS, Quantitation

## Abstract

**Purpose:**

Apolipoprotein A‐I (ApoA‐I) and apolipoprotein B‐100 (ApoB‐100) are amphipathic proteins that are strong predictors of cardiovascular disease risk. The traceable calibration of apolipoprotein assays is a persistent challenge, especially for ApoB‐100, which cannot be solubilized in purified form.

**Experimental design:**

A simultaneous quantitation method for ApoA‐I and ApoB‐100 was developed using tryptic digestion without predigestion reduction and alkylation, followed by LC separation coupled with isotope dilution MS analysis. The accuracy of the method was assured by selecting structurally exposed signature peptides, optimal choice of detergent, protein:enzyme ratio, and incubation time. Peptide calibrators were value assigned by isobaric tagging isotope dilution MS amino acid analysis.

**Results:**

The method reproducibility was validated in technical repeats of three serum samples, giving 2–3% intraday CVs (*N* = 5) and <7% interday CVs (*N* = 21). The repeated analysis of interlaboratory harmonization standards showed −1% difference for ApoA‐I and −12% for ApoB‐100 relative to the assigned value. The applicability of the method was demonstrated by repeated analysis of 24 patient samples with a wide range of total cholesterol and triglyceride levels.

**Conclusions and clinical relevance:**

The method is applicable for simultaneous analysis of ApoA‐I and ApoB‐100 in patient samples, and for characterization of serum pool calibrators for other analytical platforms.

**Abbreviations**
ApoA‐I: apolipoprotein A‐I
ApoB‐100: apolipoprotein B‐100
CVD: cardiovascular disease
IS: internal standard
SDC: sodium deoxycholate
TC: total serum cholesterol
TG: triglyceride

## 1 Introduction

Cardiovascular disease (CVD) is a progressive condition that can lead to heart attack or stroke; two major causes of mortality throughout the world. Epidemiologic studies consistently show strong correlation between plasma lipoprotein levels and CVD risk 1. Lipoproteins are relatively large endogenous molecular assembles with unique size, density, and lipid/protein composition. Lipoproteins, circulating in fasting state, are commonly classified by their density into high, low, intermediate, and very low density classes (HDL, LDL, IDL, and vLDL) 2.

**Clinical Relevance**

In the ongoing effort to improve cardiovascular risk assessment and treatment strategies, apolipoprotein (Apo) A‐I and B‐100 based metrics of plasma lipoprotein levels is gaining clinical acceptance. Currently, clinical measurements of ApoA‐I and ApoB‐100 are performed by immunoassays coupled with various indirect detection techniques that require traceability to purified or recombinant protein primary calibrators. The traceable calibration of apolipoprotein immunoassays is a persistent challenge, especially for ApoB‐100, which cannot be solubilized in purified form. The MS‐based analytical approach is a potential alternative that allows quantification of ApoA‐I and ApoB‐100 in biological matrices by proteolytic digestion and selective quantification of protein‐specific tryptic peptides in the digestion mix. This approach allows the use of external synthetic peptide calibrators that are traceable to amino acid primary reference standards. However, a major challenge of the MS‐based approach is the stoichiometric cleavage of the signature peptides from the apolipoproteins of interest. This work addresses the stoichiometric cleavage problem and demonstrates method precision and accuracy that allows the application of the method both for simultaneous analysis of ApoA‐I and ApoB‐100 in patient samples, and for characterization of serum pools that can be used as apolipoprotein calibrators for other analytical platforms.

In most healthcare settings, routine CVD risk assessments include the fasting lipid profile, HDL‐C and LDL‐C, along with total serum cholesterol (TC) and triglyceride (TG) levels. Historically, the use of the standard lipid panel has led to improved screening of high risk individuals. However, as shown in a frequently cited study of 136 905 patients admitted to 541 hospitals over a 6‐year period, it was found that about 50% of the patients had HDL‐C, LDL‐C, and TG levels in a range near normal cut‐off values at the time of admission 3. Based on standardized TC and TG metrics of lipoproteins, numerous clinical guidelines and risk calculators were developed that vary by geographical regions and ethnicity 4, 5; however, their application is often found confusing, impractical, or underused in common individual patient care settings 6.

In an effort to improve CVD risk assessment, apolipoprotein (apo) based metric of lipoproteins is gaining clinical acceptance 7. Apos are responsible for the structural integrity and function of lipoprotein particles, and are potentially more directly linked to the underlying lipid metabolism irregularities that lead to CVD in individual patients. The main apos with established CVD risk correlation are Apolipoprotein A‐I (ApoA‐I) and apolipoprotein B‐100 (ApoB‐100). As the main functional proteins of lipid particles, ApoA‐I and ApoB‐100 provide a more accurate measure of HDL and LDL particle levels than traditional TC and TG metrics.

Currently, approved clinical measurements of apos are performed by immunoassays coupled with a variety of labeling and indirect detection techniques. The calibration of immunoanalyzer platforms and kits is achieved in a hierarchical reference method system, by calibrating one affinity assay with another using value assigned serum pools as calibrators. The value assignment of the ApoA‐I calibrator pools can be traced to a specific purified ApoA‐I reference material, but the ApoB‐100 calibrator pools can be traced only to a freshly prepared LDL density fraction pool 8, 9.

LC‐MS/MS analysis of proteins is based on direct molecule mass selective detection of protein specific proteolytic peptides (target or signature peptides) generated by trypsin. The LC‐MS/MS peak area of the native target peptide is normalized with the simultaneously detected peak area of an analogous stable isotope labeled internal standard (IS), allowing the calculation of their peak area ratio (response ratio or response factor). The calibration of the LC‐MS/MS measurement is performed by generating a response ratio versus calibrator concentration curve. This approach has typically been called isotope dilution MS (IDMS). Ideally, the calibrator is a purified, recombinant protein with known molecular weight, in dry powder form that can be accurately weighed. However, such synthetic recombinant protein is not always available. Even when it is available, it cannot be measured out by weight because of uncontrollable water content, posttranslational modifications, or it cannot be solubilized from a dried isolated state; as in the case of ApoB‐100 10.

An alternative way of addressing the calibration traceability problem of apolipoproteins measurements is with the use of protein specific, synthetic proteolytic peptide analogs as external calibrators. The feasibility of the external peptide calibration IDMS approach was demonstrated for the value assignment of the purified ApoA‐I primary standard (BCR‐CRM‐393) 11, and is considered as a model for a primary reference method procedure of apos 10. The peptide calibration approach for ApoB‐100 has proven to be more challenging. Because of its size (515 kDa) and physicochemical properties, purified ApoB‐100 forms aggregates that precipitate without its phospholipid matrix 12. Solubility problems during protein digestion can be mediated with predigestion reduction and alkylation, high concentrations of various detergents or organic solvents in the digestion mix, and overnight digestion 13, 14, 15, 16, 17. These steps are often followed by postdigestion cleaning steps, such as detergent precipitation 18, SPE, 19, 20, or antipeptide antibody capture 21. However, these extensive pre‐ and postdigestion treatment steps can contribute to unintended peptide modifications, extended sample preparation times, and cleavage product degradation 22.

In this study, we explored an approach that did not use predigestion reduction/alkylation for the quantitation of ApoA‐I and ApoB‐100. The key to our approach is the use of target peptides that are rapidly cleaved by trypsin from solvent exposed, loosely packed structural domains of lipidated ApoA‐I and ApoB‐100, as embedded into HDL and LDL particles. To evaluate this approach, we performed precision tests by intraday and interday technical repeats on three serum samples, and 24 patient samples with a wide range of TC and TG levels. We also analyzed matrix‐based reference reagents (SP1‐01 and SP3‐08) that are currently accepted for standardization by the World Health Organization (WHO). Based on our results, we make a case for the use of external peptide calibration as a feasible approach for value assignment of ApoB‐100 calibrators for other analytical platforms.

## 2 Materials and methods

### 2.1 Solvents and reagents

Unless specified, all reagents and solvents were purchased from Thermo Fisher Scientific (Fair Lawn, NJ, USA). Lyophilized Rapigest SF^®^ (RSF) detergent was purchased from Waters Corporation (Milford, MA, USA). The RSF detergent was resuspended to 0.6% g/g concentration using 500 mM Tris‐HCl/1mM CaCl_2_, pH 8.5. The RSF working solution was stored for maximum 30 days at 4^○^C. Trypsin Gold (MS‐grade) was purchased from Promega Corporation (Madison, WI, USA); a 1 mg/mL trypsin solution was prepared fresh before use in 500 mM Tris‐HCl/1mM CaCl_2_, pH 8.5.

### 2.2 Preparation and value assignment of synthetic peptide stock solutions

Native and ^13^C/^15^N‐labeled synthetic peptides were purchased in solid form from Midwest Bio‐Tech (Fishers, IN). The individual peptide stock solutions were prepared with 0.1% formic acid/water and nominal concentration of 50 pmol/μL, then distributed into 400 μL aliquots and stored at −70°C. A frozen aliquot of each peptide stock solution was sent to Midwest Biotech for amino acid analysis. A second amino acid analysis of each stock solution was performed in house by four repeated measurements. The in‐house method incorporated isobaric‐tagging (iTRAQ, AB Sciex) and isotope dilution MS, using 15 NIST (U.S. National Institute of Standards and Technology) certified native amino acid calibrators and their isotope‐labeled analogs 23. The accuracy of concentrations in the reconstituted native and IS stock solutions was confirmed by experiments comparing native/IS area ratios versus native/IS expected mole ratios (Table 1).

**Table 1:** Peptides chosen for ApoA‐I and ApoB‐100 quantitation

Protein	Peptide	MRM	% Difference of amino acid analysis methods (in‐house method versus vendor method)		[Native/IS area ratio]/[native/IS mole ratio]
			Native	IS	
ApoA‐I	AELQEGAR	y6, y7, y8	0.0	+5.6	1.06
	AHVDALR	y4, y5	+7.4	+7.3	0.94
ApoB‐100	ATGVLYDYVNK	y5, y6, y7	+8.6	+6.6	1.07
	LATALSLSNK	y5, y6, y8	+5.2	+5.3	1.02

Concentrations of peptide stock solutions were determined by amino acid analysis using NIST traceable calibrators and based on value assignment by the peptide vendor. As additional evidence of peptide concentration accuracy the match between Native/IS peak area ratio versus expected mole ratios are shown.

### 2.3 Preparation of calibration standards

The value assigned frozen peptide stock solutions were thawed, mixed together, and diluted with 0.1% formic acid using a Biomek FXp liquid handler system from Beckman Coulter (Brea, CA, USA) for enhanced reproducibility. A series of working calibration standards were prepared, at nine concentration levels between 60–60 000 nmol/L for ApoA‐I and 5–1300 nmol/L for ApoB‐100, and spiked with the IS solution mix. Each working calibration standard mix also contained 1 nmol/mL Glu‐fibrinopeptide solution to reduce adsorptive peptide loss. The calibration standard/IS mix series was stored at 4°C for a maximum of 4 weeks.

### 2.4 Preparation and storage of serum samples

Three fresh units of serum from anonymous donors (∼400 mL units) were purchased from Interstate Blood Bank (Memphis, TN.). After arrival, each unit was immediately distributed into 0.5–1 mL aliquots, and stored at −80°C until use as quality control materials (QC1, QC2, and QC3). Fresh residual serum aliquots from 24 deidentified patients were provided by Health Diagnostic Laboratory Inc. (Richmond, VA); analyzed after storing in 4°C refrigerator overnight. The reference sera used for evaluating method bias were SP1‐01 (ApoA‐I) and SP3‐08 (ApoB‐100). Both reference serum standards were provided from the Lipid Standardization Program at the Centers for Disease Control and Prevention (Atlanta, GA). After thawing, the standards were distributed into smaller aliquots and stored at −80°C for interday repeat analysis. Two frozen secondary reference serum pools (blue and white cap) were also received from Northwest Lipid Research Laboratory (Seattle, WA) and stored at −80°C until use.

### 2.5 Trypsin digestion using RSF detergent

To 10 μL of sera, 990 μL of 1× PBS, pH 8.5 was added in a 1.5 mL microcentrifuge tube (Eppendorf, USA). When mentioned, samples were reduced with 5 mM dithiothreitol for 30 min at 60°C, then alkylated with 10 mM 2‐iodoacetamide for 60 min at 25°C in the dark. The digestion was performed in 8‐well 350 μL PCR sample strips (FisherScientific, USA). To each well containing 30 μL of diluted serum, 5 μL of labeled IS peptide mix, 5 μL 0.6% RSF, and 5 μL of 1 mg/mL trypsin was added followed with digestion at 37°C for 3 h. To quench the digestion and degrade the acid labile RSF detergent, 3 μL of 0.5N HCl was added, followed by a 30‐s mixing on a benchtop vortexer at 200 rpm, and incubation for 1 h at 37°C.

### 2.6 Trypsin digestion using sodium deoxycholate detergent

A 4 μL aliquot of each serum sample was diluted 50‐fold with addition of 50 mM ammonium bicarbonate, pH 8.0, IS peptide mix, and 0.5% sodium deoxycholate (SDC; Sigma, St. Louis, MO). To stop the digestion, 10 μL 20% formic acid was added precipitating the SDC. After centrifuging the samples for 2 min at 1700 rpm, the supernatant was removed manually for IDMS analysis.

### 2.7 LC‐MS/MS analysis

All analyses were performed using a 6500 QTRAP (Sciex, Framingham, MA) controlled by Analyst software (v1.6.2). The Acquity UPLC system (Waters) was used with a 2.1 × 100 mm column packed with HALO C18 stationary phase (2.7 μm particle size) flowing at 350 μL/min. Solvents A and B were 0.1% formic acid in 100% water and 0.1% formic acid in 100% ACN. The 8‐min gradient started at an A:B ratio of 98:2 and was held constant for 30 s. Over 5 min, the gradient was increased to 80:20. The gradient was then increased to 5:95 over 1 min and then held constant for 30 s. Finally, the column was reequilibrated to initial conditions over 1 min at a flowrate of 600 μL/min. The mass spectrometer was set to operate in scheduled MRM. A 60 s window was scheduled around the expected retention times with a target cycle time of 0.6 min. The source temperature was 500°C with a spray voltage of 5 kV. The collision gas setting was set to “high.”

### 2.8 Calculation of protein concentrations from peptide calibration curves

The LC‐MS/MS peak areas were measured using MultiQuant (v3.0.2, AB Sciex). A separate (area ratio) versus (mole ratio) calibration curve with 1/x weighing was generated for each peptide MRM.

(1) Area  ratio = Native  peptide  peak  area  IS  peptide  peak  area = Slope × Mole  of  native  peptide  in  standard  Mole  of  IS  peptide  in  standard + Intercept

The response ratios of the 100‐fold diluted unknowns for ApoA‐I were between the second and fourth standards, and for ApoB‐100 between the fourth and seventh standards (first being the highest standard).

Using the calibration curve slope and intercept for each MRM, protein concentrations in the unknown samples were determined by:

(2) Protein  concentration = Area  ratio  for  unknown − Intercept  Slope × Mole  of  IS  peptide  in  unknown ×1 Digested  serum  volume

All calculated concentrations were exported into JMP (v11, SAS, Cary, NC). The reported ApoA‐I and ApoB‐100 concentrations were calculated as the average of the concentrations derived from corresponding MRMs (five MRMs for ApoA‐I and six MRMs for ApoB‐100).

### 2.9 Other methods

The ApoB‐100 ELISA kit was purchased from Abcam (Cambridge, MA) and used according to the directions provided by the manufacturer (Supporting Information). Cholesterol and TG analysis was performed with an IDMS method, calibrated and validated using NIST‐certified reference materials (Supporting Information). To obtain HDL and LDL fractions and to measure HDL‐C and LDL‐C concentration, samples were fractionated by asymmetric flow field‐flow fractionation (AF4) 24. Fifty microliters aliquot of serum was injected into an AF2000 system (Postnova Analytics, Germany) with PBS buffer as the carrier fluid. HDL‐C and LDL‐C in the serum samples were calculated by summing the measured cholesterol content of the individual fractions from 6 to 18 nm and 18 to 30 nm, respectively. TC and TG of the serum fractions were also measured by IDMS analysis without AF4 fractionation. Non‐HDL‐C was calculated as (TC) − (HDL‐C).

## 3 Results

### 3.1 Peptide screening

**Figure 1:**
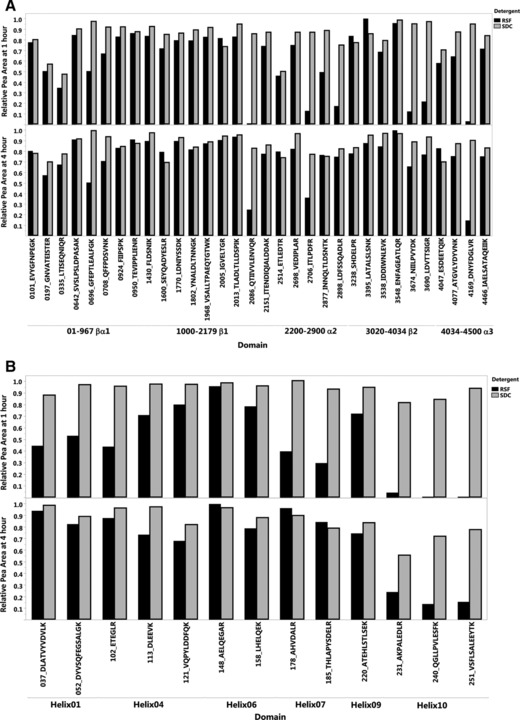
Digestion of an LDL size fraction for ApoB‐100 (A) and an HDL size fraction for ApoA‐I (B) monitoring 32 and 13 target peptides, respectively. Figure shows the digestion mix at 1 h (top) and 4 h (bottom). The full time‐course data during 24 h is presented in Supporting Information Fig. 2.

To find peptide candidates suitable for high‐throughput sample analysis without reduction and alkylation steps, discovery experiments were performed considering the entire sequence of ApoA‐I and ApoB‐100. Based on these discovery experiments (data not shown), we generated an extended list of rapidly releasable ApoB‐100 and ApoA‐I peptides. We eliminated sequences with doubly charged molecular ions >1200 *m/z* (instrument upper mass limit), and amino acid residues prone to oxidation (M and W), reducing the number of peptides to 32 for ApoB‐100 and 13 for ApoA‐I. Lipoprotein rich serum was fractionated by AF4 and fractions were merged to obtain solutions of isolated HDL and LDL. Three replicate aliquots of the HDL and LDL samples were digested with RSF and SDC detergents. At each time point during a 24‐h digestion (0.08, 0.25, 0.5, 1, 2, 4, 8 and 24 h), a 30 μL aliquot was analyzed by LC‐MS/MS (Supporting Information Figs. 1 and 2). The peak areas were normalized by the maximum peak area found for each peptide in the entire experiment (including SDC and RSF digestions). The normalized peak areas versus the peptide sequence/position at 1 and 4 h are compared in Fig. 1A and B. As shown by the 1 h peak areas, the digestion rates were faster with SDC than with RSF. However, after 8 h several peptides area counts decreased with SDC, indicating their significant degradation after cleavage.

### 3.2 Validation of maximum peptide cleavage

**Figure 2:**
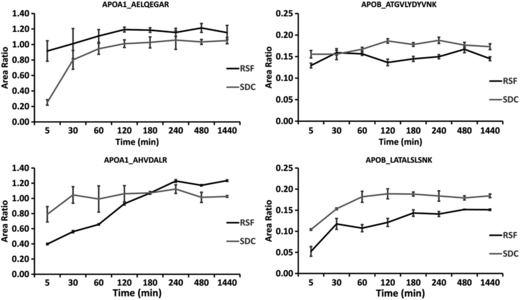
Time‐course experiment to confirm maximum peptide cleavage using 0.6% RSF and 0.5% SDC. Five replicate digestions were performed for each time point with one LC‐MS/MS injection from each replicate. With RSF detergent, maximum area ratios were reached between 3–4 h and remained near constant through 24 h. With SDC detergent, the higher maximum area ratios were the result of labeled peptide degradation as shown by the absolute native and labeled peptide areas in Supporting Information Fig. 4.

Based on stable and relatively high peak area counts between 3 and 4 h, we selected four ApoA‐I peptides and four ApoB‐100 peptides and obtained both their native‐ and isotope‐labeled analogs. The digestion time course experiment was repeated using diluted serum spiked with the labeled peptides. Using 0.6% RSF and 0.5% SDC, five replicate digestions were prepared and analyzed over 24 h, mean area count profiles are shown in Supporting Information Fig. 4. Of the labeled peptides spiked into the digestion mix at the beginning of the incubation, two showed stable peak areas for ApoA‐I and two for ApoB‐100 with the RSF detergent, decreasing <5% during the course of 24 h. With SDC, only one of the four labeled peptides remained stable both for ApoA‐I and ApoB‐100. The degradation of the ISs with SDC was also apparent from the final area ratios versus digestion time curves (Fig. 2), showing somewhat higher maximum area ratios with SDC than with RSF. Therefore, at our chosen conditions, without predigestion reduction and alkylation, RSF was found to be a more suitable detergent than SDC. Based on cleavage efficacy and stability, two ApoB‐100 peptides, LATALSLSNK‐3395 and ATGVLYDYVNK‐4077, and two ApoA‐I peptides, AELQEGAR‐148 and AHVDALR‐178, were selected for quantification; using the native analogs as calibrators and their labeled analogs as IS. After further experiments to check ruggedness with variation of pH and temperature, a 3‐h incubation time was chosen. Nontargeted MS/MS experiments showed no signs of deamidation or missed cleavage products around these sequences.

### 3.3 Validation of purity and concentration of peptide calibrators

The purity of the frozen peptide stock solutions was first checked with UPLC‐UV analysis. One frozen aliquot of each native and stable isotope labeled analog was sent to a commercial laboratory for amino acid analysis (Table 1). These initial value assignments were used to estimate the amount of reagents necessary for in‐house amino acid analysis 23. The in‐house method was quality controlled with three NIST‐certified peptides (2.7–4.2% CV range). The measurements on the ApoA‐I and ApoB‐100 peptides were performed in four parallel reactions using iTRAQ derivatization and LC‐MS/MS analysis. The peptide concentrations of the stock solutions were calculated based on multiple amino acid data (Table 1). Only the in‐house measurements were used for calculation of the calibration standards concentrations. The concentration values received from the commercial laboratory matched our in‐house measurements with >90% accuracy (Table 1). As an additional confirmation of the amino acid analysis accuracy, equal volumes of native‐ and isotope‐labeled analogs were combined and analyzed by the LC‐MS/MS method. After correction for the expected concentration, the mean peak area response ratio and expected mole ratio matched with 1.0 ± 0.04 accuracy for the ApoA‐I peptides and 1.04 ± 0.03 for the ApoB‐100 peptides (Table 1).

### 3.4 Validation of reproducibility

**Figure 3:**
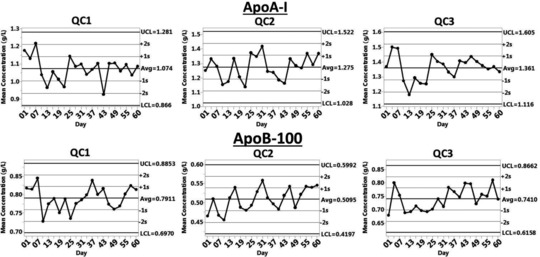
Characterization of three quality control serum pools. Coefficients of variation for intraday measurements were <2–3% (*N* = 5) and for mean interday measurements were ≤7% (*N* = 21).

The method reproducibility was assessed in three ways as follows. (i) Three control sera (QC1, QC2, and QC3) were digested and analyzed in 21 runs over a 60‐day period (*N* = 5 each day) as shown in Fig. 3 (actual values shown in Supporting Information Table 1). The intraassay quintuplicate measurements had 2–3% CVs. The interassay CVs, calculated from the 21 intraassay means, were <7% both for ApoA‐I and ApoB‐100. (ii) During the 21 method validation runs, we also analyzed reference standards (*N* = 5 each day), SP1‐01 for ApoA‐I and SP3‐08 for ApoB‐100, giving 3.9 and 4.8% CV (calculated from intraday mean values). (iii) Twenty‐four serum samples were measured by duplicate analysis on 3 days. The concentration range of the samples was 81–290 mg/dL for TC, 35–284 mg/dL for TG, 94–271 mg/dL for ApoA‐I, and 41–143 mg/dL for ApoB‐100. The average CV of the individual intraday duplicate measurements were 3.1% for TC, 4.0% for TG, 2.2% for ApoA‐I, and 3.8% ApoB‐100. The average interday CVs were 3.5, 6.5, 6.3, and 5.1%, respectively.

### 3.5 Validation of linearity and accuracy

Method linearity was tested with analysis of serum dilutions 1:200, 1:100, and 1:60 (Supporting Information Fig. 5). In equivalent concentration of the unknown samples before 1:100 dilution, these dilutions represented a range of 0.5–2.0 g/L for both ApoA‐1 and ApoB‐100. During the 60‐day method validation, the peptide calibration standard series and IS solutions were prepared two times from the original individual stock solutions, causing <2% change in the measured mean of the three QC pools; in the range of the method precision variability.

**Figure 4:**
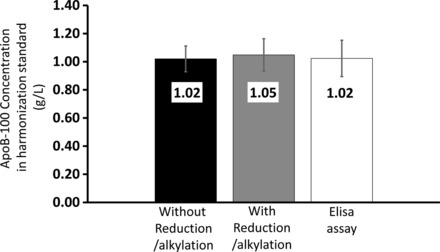
Examination of method bias by analysis of an ApoB‐100 harmonization standard. Five replicate digestions were analyzed on 3 days (*N* = 15). From left to right, the concentration of the ApoB‐100 reference standard was measured by using the RSF detergent with no reduction and alkylation (CVs = 6%), RSF detergent with reduction and alkylation (CV = 11%), and ApoB‐100 ELISA assay (CV = 19%). Error bars represent SD of measurements.

The absolute method bias was evaluated by comparison of the mean concentrations measured on 21 days in the SP1‐01 and SP3‐08 WHO standards with the assigned values. In the SP1‐01 standard, the measured mean ApoA‐I concentration was 1.49 g/L, −1% bias relative to the assigned value of 1.5 g/L. In the SP3‐08 standard, the mean ApoB‐100 concentration was 1.02 g/L with a −12% bias relative to the assigned value of 1.16 g/L. To confirm that the bias for the ApoB‐100 standard was not caused by avoiding the predigestion reduction and alkylation steps, we performed comparative analysis and found that with and without reduction and alkylation (*n* = 5 each) the bias was similar, −12 ± 0.72% and −10 ± 0.99%, respectively (Fig. 4).

In Supporting Information Fig. 6, the impact of potential bias was examined based on the data from the analysis of 24 serum samples with a wide range of TC and TG values. The between‐MRM correlation slopes for the ApoA‐I peptides were 0.97–0.98 (*R* ^2^ = 0.93–0.99), for ApoB‐100 peptides 0.91–0.97 (*R* ^2^ = 0.96–0.97). The between peptide correlations for ApoA‐I (AHVDALR/AELQEGAR) was 0.98 (*R* ^2^ = 0.91) and for ApoB‐100 (LATALSNK/ATGLVYDYVNK) was 1.07 (*R* ^2^ = 0.95). Three MRM transitions from each peptide were used for calculation of the protein concentrations, except for the ApoA‐I peptide AHVDALR using only two transitions because of an interference in the heavy labeled IS.

We also analyzed the SP3‐08 standard using a commercially available ApoB‐100 ELISA kit with vendor provided calibration standards that were harmonized with another immunoassay reference standard (SP3‐07, vendor information). The ELISA assay agreed with our IDMS measurements showing similar −10 ± 1.9% bias relative to the assigned SP3‐08 value (Fig. 4). An additional test of both linearity and accuracy was the analysis of low and high secondary reference serum pools (blue and white cap, Supporting Information Fig. 5) from Northwest Lipid Research Laboratory (Seattle, WA). For ApoA‐I, we obtained 8 and 1% bias, and for ApoB‐100 −11 and −10% bias, respectively (*N* = 5 repeats on the same day).

## 4 Discussion

### 4.1 Selection of target peptides and digestion conditions

In general, finding suitable target peptides for quantification of apolipoproteins is a challenging task because of the difficulty presented by the significant differences between quaternary structure of apolipoproteins in endogenous lipidated and denatured purified states. These structural differences also effect the solvent exposure of signature peptides and their accessibility to proteolytic cleavage by trypsin. The efficiency and rate of cleavage of specific peptides can also be very different in various digestion conditions. Therefore, the first step of our peptide selection process was the examination of peptide cleavage rates and efficacies along the protein sequence in the presence of RSF and SDC detergents. The pattern of the relative peptide cleavage rates as a function of ApoA‐I and ApoB‐100 sequence position can be compared in Fig. 1. In general, the cleavage rates were more consistent across the protein sequence with SDC than RSF. With RSF, fast trypsin cleavage correlated with flexible structural regions, in a pattern predicted by molecular dynamics models of HDL (ApoA‐I) and LDL (ApoB‐100) 2, 25, 26, 27. The stronger correspondence of cleavage rates with structural features of lipid bound ApoA‐I and ApoB‐100 suggests that RSF caused less significant denaturation of the HDL and LDL structures than SDC.

Based on cleavage and stability, we selected two peptides, ATGVLYDYVNK‐4077 and LATALSLSNK‐3395 for quantification (Fig. 2). According to the consensus model of LDL, ApoB‐100 has a pentapartite NH_2_‐βα_1_‐β_1_‐α_2_‐β_2_‐α_3_‐COOH domain structure 26. Coincidently, LATALSLSNK‐3395 is located on the β_2_ domain near the LDL receptor binding site of ApoB‐100, while ATGVLYDYVNK‐4077 is near a sharp loop turn of the α_3_ domain 26; thus expected to be surface exposed for fast trypsin cleavage. The cleavage rate patterns in Fig. 1 also indicated the solvent exposure of the βα_1_ domain, which is folded and less attached to the phospholipid surface in LDL. One of the peptides in this region, TEVIPPLIENR‐950 was also rapidly cleaved, but the sequence contains a P955S variant site (rs13306206) and was dismissed as a candidate for quantification.

ApoA‐I consists of ten amphipathic α helices 28. During the initial discovery experiments peptides from helix 2, 3, 5, 7, and 8 gave LC‐MS/MS peaks with relatively low signal to noise. The greatest difference between using SDC and RSF detergents was seen for helix 10 peptides. In the presence of RSF, the highest cleavage rates were observed around helix 6. In view of current consensus models of discoidal and spherical HDL, these findings are not surprising 2, 25, 29. Helix 10 functions as the main anchor of ApoA‐I in phospholipid surface binding. Modeling evidence suggests strong intermolecular interactions between helices 5/5’, 2/8’, and 3/7’, the main stabilizing forces in the antiparallel arrangement of ApoA‐I molecules in both discoidal and spherical HDL 2, 25, 29; Helix 5 is also recognized as the lecithin cholesterol acyl transferase binding site 2. The models also predict relatively loose 4/6’ and 6/4’ helix/helix alignments on the two sides of 5/5’ alignment. Coincidently, our selection process led to target peptides from the flexible helix 6 region, AELQEGAR‐148 and AHVDALR‐178, consistent with their fast trypsin cleavage.

It is important to note that the observed cleavage rates of the ApoA‐I and ApoB‐100 peptides were the result of our digestion strategy without reduction and alkylation. Other research groups chose to use predigestion reduction and alkylation steps and observed different cleavage rate patterns, therefore, they arrived to a different set of signature peptides for ApoA‐I and ApoB‐100 quantification 13, 14, 15, 16, 18, 21. In general, apolipoprotein surface exposed regions occur because of proline and glycine rich loop regions. Because the RSF detergent caused only partial denaturation of the protein structures, our selected signature peptides are consistent with the native lipidated apolipoprotein structures. Coincidentally, polar proline containing peptides often elute in faster and sharper peaks and at lower organic eluent content on reverse phase columns. They also ionize well in the ESI LC‐MS interface. Therefore, our digestion strategy and the LC‐MS detection technique naturally led us to the selection of short, polar ApoA‐I and ApoB‐100 signature peptides.

We chose RSF detergent for two main reasons. First, the observation that ApoA‐I and ApoB‐100 maintained their lipid bound tertiary structure in the presence of RSF was thought to be an advantage by improving consistent exposure of the selected target peptides and resulting in more reproducible digestion rates and cleavage recovery. Second, RSF allowed a simpler sample preparation workflow that was more feasible for automation. RSF by design is an acid‐labile detergent that can be degraded by simple addition of acid. Although the time course experiments showed that RSF and SDC worked with similar digestion efficacy, the acidification after digestion with SDC generated a significant amount of precipitate that was difficult to remove in an automated fashion.

The digestion efficiency was also determined by the trypsin:protein ratio, 5 μg trypsin for 18–25 μg protein in 0.3 μL serum (assuming 60–80 g/L of protein in a typical human serum sample and digesting 30 μL of 100× diluted serum). In general, we found that in various apolipoprotein studies using a high amount of trypsin is very common (3–10 μg/sample) 18, 21. In our method, using 1:4 to 1:5 trypsin:protein ratio and relatively small volume of serum also allowed the elimination of common pre‐ and postdigestion steps found in traditional proteomic workflows (i.e., reduction, alkylation, extraction), shortened the sample processing time, and led to significant savings by not using other reagents and consumables.

Altogether, the selection of rapidly cleaved target peptides, small amount of serum (0.3 μL), high trypsin:protein ratio allowed a short sample preparation time of 4 h. Because there was less time for cleavage product and IS peptide degradation, avoiding the predigestion alkylation step did not affect accuracy and even enhanced the method precision.

In comparison to other reported IDMS methods, our digestion and peptide calibration based approach has the disadvantage of being limited to the simultaneous analysis of only ApoA‐I and ApoB‐100. This is mainly because the steep criteria of the peptide calibration based quantification. The calculation of accurate protein concentrations based on the measured cleavage product concentration is possible only if the digestion method is optimized to the maximum cleavage of the target peptides from the protein; approaching stoichiometric cleavage efficiency as much as possible. With increasing the number of proteins and corresponding peptide targets, the level of difficulty for meeting this criteria increases substantially. Therefore, we concede that with the peptide‐based calibration approach the level of multiplexing is more limited than with protein‐based calibration, where the bias from less than stoichiometric cleavage is automatically corrected by the protein calibration curve. In our laboratory, we use the peptide‐calibrated IDMS approach only for ApoA‐I and ApoB‐100. We also used this method to characterize calibrator pools for other platforms such as on‐line trypsin digestion coupled LC‐MS/MS where we apply protein calibration and higher level of multiplexing 30.

### 4.2 Peptide based IDMS method accuracy

The absolute accuracy and precision of the ApoA‐I and ApoB‐100 serum concentration measurement was assured by taking multiple provisions. First, we obtained evidence of complete peptide cleavage, that is, confirmation of limit peptide behavior 31, 32, by showing that the native/IS response ratios remained constant while IS signal intensities remained stable. Second, extra steps were taken for the value assignment of the peptide stock solutions by an amino acid analysis method with enhanced specificity and reproducibility with NIST‐certified amino acid standards. Third, the peptide calibration series was prepared using a carefully volume calibrated liquid handler. Fourth, we used matrix‐matched calibration to minimize LC‐MS/MS analysis variability, that is, the peptide calibration standard series was incubated at the same time with the protein samples in the presence of the same reagent mix. Fifth, to further minimize matrix effects, we diluted the serum samples 100‐fold, the maximum fold dilution that still allowed sensitive LC‐MS/MS detection. Sixth, the average of multiple MS fragment ions was used for each concentration determination. Seventh, the average of two peptide concentrations was used for the calculation of the protein concentrations. Eighth, three quality controls were used to monitor method reproducibility, all three resulting in <3% intraassay CVs and <7% interassay CVs.

Based on the average of multiple measurements on 21 days, our method compared well (−1% bias) with the ApoA‐I WHO reference standard (SP1‐01). For the ApoB‐100 WHO standard (SP3‐08), our method showed a −12% bias. We note that the value assignment of these reference standards was made around 1990, with the best quality of technologies available at that time. The value assignment of the SP1‐01 standard was performed by RIA using a primary purified reference standard (BCR‐CRM‐393), value assigned by amino acid analysis,^8^ and later by HPLC‐coupled IDMS 11. The value assignment of the SP3‐08 standard was performed by immunonephelometry with purified LDL used as an apoB‐100 calibrator, which was measured by an SDS‐Lowry procedure calibrated with purified BSA 9. Therefore, the ApoB‐100 value assignment of SP3‐08 was performed without a direct primary measurement procedure or primary reference material 10. There is still no primary reference material available for ApoB‐100 because purified protein materials that are commutable have not yet been produced. It also should be noted that these reference materials were always intended to be an interim solution until more accurately value‐assigned primary reference materials and an absolute accuracy‐based primary reference measurement procedure is developed 10.

### 4.3 Correlation of lipid levels with ApoB‐100 and ApoA‐I levels

**Figure 5:**
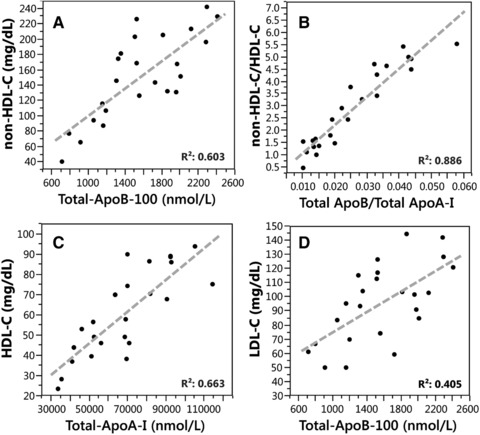
Correlation between cholesterol and apolipoprotein concentrations for 24 serum samples. (A) Non‐HDL‐C versus ApoB‐100; (B) non‐HDL‐C/HDL‐C versus ApoB‐100/ApoA‐I; (C) HDL‐C versus ApoA‐I; (D) LDL‐C versus ApoB‐100.

In spite of their potential as strong risk factors, apo analysis has not been generally translated from research to regular diagnostic use for CVD risk assessment; and is only considered as an advanced test. The current clinical guidelines still advocate the use of lipid level based measures such as non‐HDL‐C (non‐HDL‐C = TC − HDL‐C), which can be readily implemented in clinical settings without additional cost. The correlation of non‐HDL‐C with ApoB‐100 is usually found 0.8–0.85 33, 34, 35. However, the general correlation of calculated lipid measures with ApoB‐100 levels may not result in similar concordance with risk. In patient groups with wide range of non‐HDL‐C levels (147‐213 nmol/L), risk classification based on non‐HDL‐C and ApoB‐100 levels agreed in less than 50% of patients 35. Our analysis of 24 patient samples with wide range of lipid profiles also showed correlation of 0.8 between non‐HDL‐C and ApoB‐100, but only for samples with <145 mg/dL non‐HDL‐C; including all samples the correlation was only 0.60 (Fig. 5A).

Another advocated lipid‐based measure is the non‐HDL‐C/HDL‐C ratio 33, 34, 35. As with previous studies, we also found similar correlation of 0.89 between the non‐HDL‐C/HDL‐C and ApoB‐100/ApoA‐I ratios (Fig. 5B). At the same time, HDL‐C versus ApoA‐I (Fig. 5C) and LDL‐C versus ApoB‐100 (Fig. 5D) correlations were relatively low, 0.66 and 0.40, respectively, indicating lack of concordance with underlying metabolic abnormalities.

## 5 Conclusions

We have developed a high throughput, absolute protein quantification IDMS work flow for the analysis of ApoA‐I and ApoB‐100 in serum that meets the accuracy and reproducibility requirements of clinical laboratory guidelines. To achieve method accuracy and precision, we followed established IDMS guidelines 36 stressing the importance of complete peptide cleavage, validation of precision based on multiple quality control serum samples, matrix‐matched peptide calibration, and NIST traceable value assignment of the peptide calibrators. An additional important outcome of this work is the evaluation of bias for a peptide calibration based IDMS method, based on the analysis of harmonization standards (serum pools) that are currently recognized as the universal reference materials for ApoA‐I and ApoB‐100 immunoassay measurements. In the IDMS application presented here, using peptide calibrators and following IDMS procedure guidelines, the bias for ApoA‐I (SP1‐01 traceable to purified ApoA‐I primary reference material) was −1%. For ApoB‐100 (SP3‐08, for which there is no purified primary reference material), the IDMS approach yielded a −12% bias (as compared to the value assignment by radial immunoassay, calibrated with materials that were value assigned by SDS‐Lowery). A −12% bias is high by clinical laboratory standards and needs further examination. For example, the assumption that in the LDL density faction, which was used as a “pseudo‐primary calibrator,” the only protein present was ApoB‐100 should be reexamined, by using modern techniques that were not available in 1990 when the SP3‐08 standard was prepared 9.

In order for IDMS measurements of ApoA‐I and ApoB‐100 to be applicable to clinical decision making or assessment of CVD risk, it is becoming increasingly important to place all measurements on a common accuracy base with clinical immunoassays. There is currently no broad consensus on the best way to implement this goal. One strategy is to correct IDMS measurements to match the value of the old ApoA‐I and ApoB‐100 immunoassay harmonization standards. For ApoB‐100 specifically, it should be recognized that the value assignment of the immunoassay harmonization standard was made without an absolute accuracy‐based reference measurement procedure and without a primary reference material 10. Our work using the peptide calibration based IDMS approach supports the notion that the uncertainties about the 100% digestion efficacy are outweighed by the systematic and stoichiometric traceability to amino acid standards. As research continues to identify the roles of apolipoprotein concentrations, particle size and particle number in CVD risk assessment, the need for accuracy‐based reference materials and reference measurement procedures will surely grow. IDMS methods calibrated with accurately traceable peptide calibrators should play a role in these efforts.

*The authors declare no conflicts of interest*.

## Supporting information

Supplementary Information
