# Supplementary material for: High throughput quantification of apolipoproteins A‐I and B‐100 by isotope dilution MS targeting fast trypsin releasable peptides without reduction and alkylation
Source: Proteomics Clin Appl. 2017 Apr 3;11(7-8):1600128. doi: 10.1002/prca.201600128 (PMC5637893; doi:10.1002/prca.201600128)
Supplement: Supplementary file 1 — Supplementary Information [file PRCA-11-na-s001.docx]

**Supplementary Information**

**Cholesterol and triglyceride analysis.** A direct LC-MS/MS method was developed for analysis of cholesterol and triglyceride in AF4 fractions and diluted human serum [Gardner et. al., in preparation]. The calibration standard mix was prepared in isopropanol. Total cholesterol was quantified as the sum of free cholesterol and cholesteryl esters, using free cholesterol and cholesteryl palmitate as calibrator, respectively. Total triglycerides were measured using triolein, tripalmitin, and trilinolein in a ratio of 3.0:1.8:1 by weight. Internal standard (IS) mix was prepared in ethanol, containing 0.033 mg/dL d7-cholesterol (for FC quantitation), 0.098 mg/dL cholesteryl-d7-palmitate (for CE quantitation), and 0.125 mg/dL d98-tripalmitin (for TG quantitation). 50µL aliquots were transferred from serum samples diluted 1:100 with phosphate buffered saline (PBS) into a 96-well plate. The protein-precipitation/extraction, by the addition of 0.2 mL IS/ethanol mix, was followed by evaporation and reconstitution in 100 µL nonane. The plate was covered with a heat-sealing foil mat for analysis by UHPLC-MS/MS. The analysis method used normal phase liquid chromatography separation and in-source collision-induced dissociation (CID) coupled with tandem mass spectrometry detection. The UHPLC system was a UHPLC-SA (Spark-Holland, Emmen, Netherlands). The mass spectrometer was a Sciex 4000 QTrap (AB Sciex, Framingham, MA). From each sample, 2µL is injected (full loop). The column was a Luna HILIC 3µm, 2x50mm (Phenomenex, Torrance, CA). Mobile phase A was hexanes with 0.1% isopropanol. Mobile phase B was 50:45:5 hexanes:ethanol:methanol. The mobile phase flow rate was 600 µL / min with gradient elution. The gradient started at 0% B, holding for 0.5 minute, then to 15% B over 0.5 minute, holding for 0.5 minute, returning to 0% B over 0.1 minute, holding 0% B for 1.9 minutes. The Heated Nebulizer (APCI) source is installed. The source conditions for all time periods in the method are: Curtain Gas = 10 psi nitrogen, Nebulizer Current = 4µA, Temperature = 325°C, Gas 1 = 70 psi nitrogen, CAD = “Medium”. MRM conditions are given in Table S2. The free cholesterol and cholesteryl esters eluted in two separate chromatographic peaks but in the same m/z MRM trace, while all the triglycerides eluted in one single peak in the same m/z MRM trace. The total cholesterol concentrations in mg/dL were calculated as a sum of the measured free cholesterol and the free-cholesterol-equivalent of all cholesteryl esters. Triglyceride concentrations were calculated in mg/dL triolein-equivalent concentration.

**ELISA assay**. The calibration series was prepared from the lyophilized purified human Apo B standard provided by the vendor (Abcam, Cambridge, MA). All samples including the calibration curve was analyzed in duplicates. After allowing the capture and detector antibodies to bind the ApoB-100 in the samples, the antibody-ApoB-100 complex was immobilized on the anti-tag antibody coated wells. The wells were washed to be remove any unbound ApoB-100. 3,3',5,5'-tetramethylbenzidine (TMB) substrate is used to stain the antibody complex. The reaction was completed by adding Stop Solution. The reaction color changed from blue to yellow. The intensity was measured using a spectrophotometer at 450 nm. The concentration of Apo B is proportional to the color produced after the reaction is completed.

**Asymmetric Flow Field-Flow Fractionation.** Asymmetric flow field-flow fractionation (AF4) is based on the fundamental nature of laminar flow. A liquid medium passes through a thin channel which causes it to adopt a parabolic velocity profile across the height of the channel, with stream velocities faster at the center of the channel and slower near the walls. When serum samples are injected into this stream, contained particles are subjected to a second, perpendicular field created by withdrawal of carrier fluid through a semipermeable membrane that drives them toward the accumulation wall, where they experience slower flow rates. Once they are concentrated at the accumulation wall, particle species begin to diffuse up away from the accumulation wall via Brownian motion into higher velocity flow regimes, with frequency and magnitude according to their intrinsic diffusion coefficient and Stokes diameter. Differential retention is caused by the different average height achieved by particles of different sizes, with smaller particles having a higher average height which results in eluting them faster. The AF4 separation requires no filtration or other sample pretreatment and is achieved by gentle fluid dynamics; both of these elements minimize the risk of introducing composition artifacts during separation.

**Figure S1.** Full time-course digestion of LDL for ApoB-100 monitored based on 32 target peptides sampling the digestion mix at 0.08, 0.25, 0.5 1, 2, 4, 8 and 24 h. Curves were normalized to the maximum peak area for each peptide during the experiment.

**Figure S2.** Full time-course digestion of HDL for ApoA-I monitored based on 13 target peptides sampling the digestion mix at 0.08, 0.25, 0.5 1, 2, 4, 8 and 24 h. Curves were normalized to the maximum peak area for each peptide during the experiment.

**Figure S3.** Correlation of lipid parameters to apolipoprotein concentrations for 25 serum samples: A) Non-HDL-C/HDL-C *vs.* ApoB-100/ApoA-I; B) Non-HDL-C LDL-C (red) and HDL-C (blue) vs. ApoA-I and ApoB-100 (marker size by ApoA-I/ApoB-100 ratios). (colors blue-grey-red by low-medium-high ApoA-I/ApoB-100 ratios). Samples were stratified into Low and High total cholesterol groups (81-210 mg/dL and 210-290 mg/dL) and total triglyceride groups (35-145 mg/dL and 145-284 ng/dL).

**Figure S4:** Time course experimental data for the quantitation peptides used for area ratio plots in Figure 2.

.

**Table S1:** Raw data values for Figure 3.

**Supplementary Figure S5:** Linearity of method shown for sample dilutions 1:200,1:100 and 1:60. The method brackets the reference ranges for ApoA-1 (a) and ApoB-100 (b). Secondary reference materials were also analyzed (c,d) for testing method accuracy. ApoA-I accepted values ranged from 1.1-1.6 g/L. ApoB-100 accepted values ranged from 0.7-1.7 g/L.

**Supplementary Figure S6:** Validation data examining peptide bias for ApoA-I and ApoB-100. For ApoA1, two transitions were chosen for the peptides AELQEGAR (a) and AHVDALR (b). The peptide average has also been displayed (c). Data for ApoB-100 is displayed in the same manner for peptides ATGVLYDYVNK (d), LATALSLSNK (e) and the peptide average (f).
